# Lean or diabetic subtypes predict increased all-cause and disease-specific mortality in metabolic-associated fatty liver disease

**DOI:** 10.1186/s12916-022-02716-3

**Published:** 2023-01-04

**Authors:** Goh Eun Chung, Su Jong Yu, Jeong-Ju Yoo, Yuri Cho, Kyu-na Lee, Dong Wook Shin, Donghee Kim, Yoon Jun Kim, Jung-Hwan Yoon, Kyungdo Han, Eun Ju Cho

**Affiliations:** 1 Department of Internal Medicine and Healthcare Research Institute, Seoul National University Hospital Healthcare System Gangnam Center, Seoul, Republic of Korea; 2 Department of Internal Medicine and Liver Research Institute, Seoul National University College of Medicine, 101 Daehak-No, Jongno-Gu, Seoul, 03080 Republic of Korea; 3 Department of Gastroenterology and Hepatology, Soonchunhyang University Bucheon Hospital, Bucheon, Gyeonggi-Do Republic of Korea; 4 Center for Liver and Pancreatobiliary Cancer, National Cancer Center, Goyang, Republic of Korea; 5 Department of Biomedicine and Health Science, Catholic University, Seoul, Republic of Korea; 6 Department of Family Medicine and Supportive Care Center, Samsung Medical Center, Sungkyunkwan University School of Medicine, Seoul, Republic of Korea; 7 Department of Clinical Research Design and Evaluation and Department of Digital Health, Samsung Advanced Institute for Health Science, Seoul, Republic of Korea; 8 Division of Gastroenterology and Hepatology, Stanford University School of Medicine, Stanford, CA USA; 9 Department of Statistics and Actuarial Science, Soongsil University, Seoul, Republic of Korea

**Keywords:** Metabolic dysfunction, Mortality, Lean, Steatosis

## Abstract

**Background:**

Metabolic-associated fatty liver disease (MAFLD) encompasses diverse disease groups with potentially heterogeneous clinical outcomes. We investigated the risk of all-cause and disease-specific mortality in MAFLD subgroups.

**Methods:**

Using the Korean National Health Insurance Service database, participants were divided into four subgroups: no MAFLD, MAFLD-diabetes, MAFLD-overweight/obese, and MAFLD-lean. Hazard ratios (HRs) and 95% confidence interval (CI) values for all-cause and disease-specific mortality according to MAFLD subgroups were analyzed using Cox proportional hazards models.

**Results:**

Among 9,935,314 participants, those with MAFLD-diabetes showed the highest risk of all-cause and disease-specific mortality. The HRs (95% CI) for all-cause mortality were 1.61 (1.59–1.63), 1.36 (1.34–1.38), and 1.19 (1.18–1.20) in the MAFLD-diabetes, MAFLD-lean, and MAFLD-overweight/obese groups, respectively. The magnitude of cardiovascular disease and cancer-related risk showed the same pattern. The risk of liver-related mortality in the MAFLD-lean group (HR: 2.84, 95% CI: 2.72–2.97) was comparable with that in the MAFLD-diabetes group (HR: 2.85, 95% CI: 2.75–2.95). When stratified by body mass index, liver-related mortality was the highest in MAFLD-lean individuals in the underweight group (HR, 5.03, 95% CI: 4.23–5.97).

**Conclusions:**

The MAFLD-lean and MAFLD-diabetes groups had a higher risk of all-cause and disease-specific mortality than did the MAFLD-overweight/obese group. Classifying MAFLD subgroups based on metabolic phenotypes might help risk stratification of patients with MAFLD.

**Supplementary Information:**

The online version contains supplementary material available at 10.1186/s12916-022-02716-3.

## Background

Metabolic dysfunction-associated fatty liver disease (MAFLD) is a new conceptual definition of fatty liver disease that is based on metabolic abnormalities (regardless of the etiology of chronic liver disease) that emphasizes the role of metabolic dysfunction in the clinical outcomes of patients with hepatic steatosis [1, 2]. MAFLD is defined according to the presence of hepatic steatosis along with one or more of the following criteria: (i) overweight or obesity, (ii) diabetes mellitus (DM), or (iii) ≥ 2 metabolic abnormalities [3]. Recent studies have investigated the predictive role of MAFLD in cardiovascular disease (CVD) [4, 5] and all-cause mortality [6–8].

However, MAFLD includes a heterogeneous group of patients, and the following subgroups should be recognized: lean and non-lean patients, alcoholic and nonalcoholic patients, and patients with isolated hepatic steatosis (vs. those with underlying liver disease) [9]. A previous study reported that MAFLDs are heterogeneous in terms of mortality outcomes and cardiovascular risk and that these outcomes vary according to the accompanying metabolic dysfunctions [10]. A previous study demonstrated that MAFLD better identifies cardiovascular risk than nonalcoholic fatty liver disease (NAFLD), which was attributed to the inclusion criteria of metabolic dysfunctions [11]. Moreover, a statistically significant difference in the degree of liver fibrosis has been reported among MAFLD subgroups, suggesting differential risks of advanced liver disease across MAFLD subgroups [12].

Since overweight/obesity is typically defined according to body mass index (BMI), it does not always entail metabolic abnormalities [13]. As MAFLD is a spectrum encompassing diverse disease groups defined according to metabolic abnormalities and overweight/obesity, there may be heterogeneity in clinical outcomes according to disease subcategorizations. Therefore, we aimed to investigate the differential risk of all-cause and disease-specific mortality according to MAFLD subgroups divided by metabolic risk factors and overweight/obesity status within a nationally representative Korean study population.

## Methods

### Data source

The present study obtained data from the Korean National Health Insurance System (NHIS). This is a national insurer managed by the Korean government, and approximately 97% of the Korean population subscribe to it [14]. The NHIS database contains health records, including sociodemographic data (age, sex, income level), anthropometric measurements, laboratory tests (e.g., lipid profiles, blood glucose levels), lifestyle behaviors (smoking, alcohol consumption, regular exercise), medical diagnoses (based on the 10^th^ revision of the International Classification of Diseases [ICD-10]), and treatment data [15]. This database has been widely used for conducting previous epidemiologic studies [16, 17].

### Study sample

This investigation included 10,585,844 adults aged ≥ 20 who underwent health screening examinations between January 1, 2009, and December 31, 2009. We ascertained outcome events after a lag of 1 year; those with outcome events within 1 year were excluded (*n* = 28,478). After excluding participants with incomplete information (*n* = 622,052), 9,935,314 subjects were analyzed.

The study protocol was approved by the Institutional Review Board of Soongsil University (SSU-202007-HR-236–01). This work conformed to the ethical guidelines delineated within the Declaration of Helsinki and its later amendments. The requirement for patient informed consent was waived as this was a retrospective study conducted exclusively using de-identified secondary data.

### Measurement of hepatic steatosis

Although ultrasonography is a first-line screening technique used in clinical practice [18], it is not included in the NHIS mass screening program. Therefore, the fatty liver index (FLI), as a surrogate marker of hepatic steatosis, was used to assess hepatic steatosis. The calculation of FLI was based on the following equation, using BMI, waist circumference (WC), triglyceride, and gamma-glutamyl transferase (GGT) data [19]. The lower cut-off of FLI ≥ 30 was used in this study [4].

$$\mathrm{FLI }=\left[\left({\mathrm{e}}^{0.953 \times \mathrm{ ln\ triglyceride }+ 0.139 \times \mathrm{ BMI }+ 0.718 \times \mathrm{ ln\ GGT}\left[\mathrm{gamma}-\mathrm{glutamyl\ transferase}\right]+ 0.053 \times \mathrm{ WC}\left[\mathrm{waist\ circumference}\right]- 15.745}\right)/ (1 + {\mathrm{e}}^{0.953 \times \mathrm{ ln\ triglyceride }+ 0.139 \times \mathrm{ BMI }+ 0.718 \times \mathrm{ ln\ GGT }+ 0.053 \times \mathrm{ WC}-15.745})\right] \times 100$$

### Definitions of MAFLD and subgroups

MAFLD was defined as the presence of metabolic risk factors in hepatic steatosis, not excluding other concomitant liver diseases and significant alcohol consumption, based on diagnostic criteria proposed in an international expert consensus statement in 2020 [3]. MAFLD was diagnosed as the presence of hepatic steatosis and one or more of the following criteria: (i) overweight or obesity (BMI ≥ 23 kg/m^2^), (ii) DM, and (iii) ≥ 2 metabolic abnormalities. The cut-off value of BMI for overweight and obesity was determined based on the criteria for the Asia–Pacific region. Metabolic abnormalities were as follows: (i) WC of ≥ 90 cm for men and ≥ 80 cm for women, (ii) blood pressure ≥ 130/85 mmHg or specific drug treatment for elevated blood pressure, (iii) fasting plasma triglycerides ≥ 150 mg/dL or specific drug treatment for elevated triglycerides, (iv) plasma high-density lipoprotein-cholesterol < 40 mg/dL for men and < 50 mg/dL for women or specific drug treatment for high cholesterol, and (v) fasting glucose ≥ 100 mg/dL. As the homeostasis model assessment of insulin resistance scores and high-sensitivity C-reactive protein levels were not available through the NHIS screening program, these criteria were not implemented in this study.

**Fig. 1:**
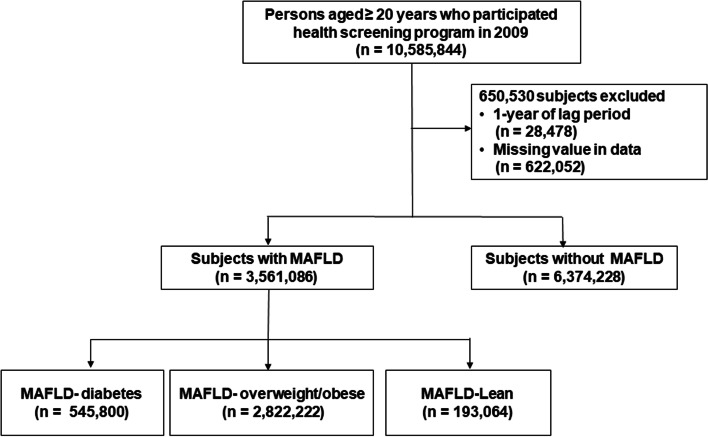
Flow chart of the study population enrollment. BMI, body mass index; DM, diabetes mellitus; MAFLD, metabolic-associated fatty liver disease

In the present study, participants identified as affected by MAFLD were classified into three groups, as described previously [12, 20]. First, we determined the diabetic MAFLD group based on the presence of DM regardless of BMI; subsequently, in subjects without DM, we classified MAFLD subgroups according to BMI: (i) overweight/obese (BMI ≥ 23 kg/m^2^) or (ii) lean or normal weight (BMI < 23 kg/m^2^). Finally, the study population was divided into four subgroups: no MAFLD, MAFLD-diabetes, MAFLD-overweight/obese, and MAFLD-lean (Fig. 1).

### Study outcomes

The primary outcomes evaluated in this study were all-cause and disease-specific mortality. Information on mortality and cause of death was available for all subjects in the cohort; cause-of-death variables were classified according to the Korean Standard Classification of Diseases and Causes of Death, based on the ICD-10 (with data provided by the Korean National Statistical Office) [21].

Based on the ICD-10 diagnostic codes, the cause of death was categorized into CVD mortality (I00-I99), cancer mortality other than hepatocellular carcinoma (C00-C97, except for C22), and liver-associated mortality (K70-76, C22) [22]. The study population was followed from baseline to the date of death or until December 31, 2019, allowing for a minimum of 12 months of follow-up for each individual.

### Covariates

As described previously [23], standardized self-reported questionnaires were used to collect data during enrollment. Briefly, smoking status (non-smokers, ex-smokers, current smokers), alcohol consumption (none, mild, heavy [≥ 30 g for males and ≥ 20 g for females per day]), and regular exercise were evaluated using a self-administered questionnaire. When participants exercised at a high intensity at least three times per week or at a moderate intensity at least five times per week, they were defined as undertaking regular physical exercise. Household income was dichotomized at the lowest 20%. Comorbidities were defined using ICD-10 diagnosis codes, prescription information in the year prior to the health screening, and health screening results. Criteria for hypertension were I10–13 or I15 claim codes in addition to ≥ 1 prescription for an antihypertensive agent or a systolic/diastolic blood pressure reading of ≥ 140/90 mmHg. Criteria for diabetes were E11–14 claim codes in addition to ≥ 1 prescription for an antidiabetic medication per year or a fasting glucose level of ≥ 126 mg/dL. The criteria for dyslipidemia were the E78 claim code and ≥ 1 prescription for a lipid-lowering agent or a total cholesterol level of ≥ 240 mg/dL. The Charlson Comorbidity Index (CCI) [24] was determined from claims data during the period spanning 2 years prior to baseline. K74 and B15–B19 claim codes were the criteria used to determine liver cirrhosis and viral hepatitis.

Height, weight, and WC were measured and evaluated by healthcare professionals during nationally mandated health screenings. BMI was calculated as the participant’s weight in kilograms divided by their height in meters squared. Blood specimens were obtained from each participant after an overnight fast. The estimated glomerular filtration rate (eGFR) was calculated from serum creatinine using an equation determined by the Modification of Diet in Renal Disease Study [25].

### Statistical analyses

Data are presented as means ± standard deviations for normally distributed continuous variables and as proportions for categorical variables (unless otherwise indicated). Comparisons of baseline characteristics were conducted using independent t-tests and analysis of variance for continuous variables and chi-square tests for categorical variables.

All-cause and disease-specific mortality rates were calculated by dividing the number of incident cases by the total follow-up period and were presented as rates per 1000 person-years. Hazard ratios (HRs) and 95% confidence interval (CI) values for all-cause and disease-specific mortality according to MAFLD subgroups were analyzed using Cox proportional hazards models. We selected potential prognostic factors a priori based on clinical relevance and a comprehensive literature review; potential prognostic factors included age, sex, BMI, income, smoking, alcohol consumption, regular exercise, and CCI scores. Statistical analyses were performed using SAS version 9.4 (SAS Institute, Cary, NC, USA). Two-sided *P*-values of < 0.05 were considered the threshold for statistical significance.

## Results

### Baseline characteristics

This analysis included 9,935,314 participants (median age [interquartile range] 46 [36–57] years; mean age, 47.2 years; 56.0% males). The prevalence of MAFLD was 35.8%. The prevalence of each MAFLD subtype was 5.5% (MAFLD-diabetes), 28.4% (MAFLD-overweight/obese), and 1.9% (MAFLD-lean). The baseline characteristics for the study population are shown in Table 1. Compared with the no MAFLD group, people with MAFLD were more likely to be male and current smokers and had higher alcohol consumption (*P* < 0.001 for all). Additionally, subjects in the MAFLD-diabetes group and the MAFLD-lean group were more likely to have hypertension and dyslipidemia than those in the no MAFLD group (*P* < 0.001). Most anthropometric and laboratory variables (including systolic/diastolic blood pressure, fasting glucose, total cholesterol, triglycerides, and eGFR) were less metabolically favorable in the MAFLD-diabetes and MAFLD-lean groups than in the no MAFLD group (all *P* < 0.001).

**Table 1:** Baseline characteristics of the study population

	No MAFLD	MAFLD-diabetes	MAFLD-overweight/obese	MAFLD-lean	*p*-value
	(*n* = 6,374,228)	(*n* = 545,800)	(*n* = 2,822,222)	(*n* = 193,064)	
Age	46.1 ± 14.4	56.4 ± 11.8	47.8 ± 13.0	51.5 ± 13.0	< .0001
Male	2,741,020 (43.0)	377,504 (69.2)	2,141,255 (75.9)	163,052 (84.5)	< .0001
Income_low	1,048,999 (16.5)	90,902 (16.7)	366,269 (13.0)	28,459 (14.7)	< .0001
Smoking					< .0001
None	4,357,567 (68.4)	268,868 (49.3)	1,233.006 (43.7)	62,161 (32.2)	
Ex	707,242 (11.1)	111,743(20.5)	572,195 (20.3)	36,044 (18.7)	
Current	1,309,419 (20.5)	165,189 (30.3)	1,017,021 (36.0)	94, 859 (49.3)	
Alcohol consumption					< .0001
None	3,667,279 (57.5)	271,833 (49.8)	1,140,952 (40.4)	57,875 (30.0)	
Mild	2,356,423 (37.0)	199,736 (36.6)	1,299,773 (46.1)	94,239 (48.8)	
Heavy	350,526 (5.5)	74,231 (13.6)	381,497 (13.5)	40,950 (21.2)	
Regular exercise	1,121,607 (17.6)	113,078 (20.7)	517,507(18.3)	30,850 (16.0)	< .0001
Diabetes	328,428 (5.2)	545,800 (100)	0 (0)	0 (0)	< .0001
Hypertension	1,156,210 (18.1)	339,728 (62.2)	1,007,110 (35.7)	77,208 (40.0)	< .0001
Dyslipidemia	793,083 (12.4)	242,146 (44.4)	716,137(25.4)	53,214 (27.6)	< .0001
Hepatitis	150,803 (2.4)	21,903 (4.0)	75,393 (2.7)	5160 (2.7)	< .0001
Liver cirrhosis	14,699 (0.2)	4641 (0.9)	8086 (0.3)	1134 (0.6)	< .0001
CCI score					< .0001
0	4,207,758 (66.0)	148,570 (27.2)	1,832,368 (64.9)	123,264 (63.9)	
1	1,206,091 (18.9)	125,506 (23.0)	577,483 (20.5)	39,673 (20.6)	
≥ 2	960,379 (15.1)	271,724 (49.8)	412,371 (14.6)	30,127 (15.6)	
BMI	22.2 ± 2.4	26.4 ± 3.1	26.7 ± 3.4	21.9 ± 1.0	< .0001
WC	75.8 ± 7.1	89.4 ± 7.9	88.5 ± 7.2	81.5 ± 10.0	< .0001
SBP	119.3 ± 14.4	131.0 ± 15.7	127.5 ± 14.3	129.5 ± 15.0	< .0001
DBP	74.3 ± 9.6	80.6 ± 10.2	79.8 ± 9.8	80.8 ± 10.0	< .0001
Glucose	93.6 ± 19.0	149.6 ± 49.9	95.6 ± 12.0	98.3 ± 12.6	< .0001
Total Cholesterol	189.2 ± 39.0	203.1 ± 48.9	206.8 ± 42.1	205.9 ± 47.1	< .0001
HDL-C	59.0 ± 31.1	51.2 ± 35.2	51.9 ± 33.64	55.7 ± 53.7	< .0001
eGFR	88.6 ± 44.4	83.3 ± 37.2	86.1 ± 48.5	87.1 ± 41.8	< .0001
TG^a^	87.6 (87.6–87.6)	188.8 (189.5–190.1)	171.6 (171.5–171.7)	232.5 (232.1–233.0)	< .0001
AST^a^	21.6 (21.6–21.6)	28.6 (28.6–28.7)	26.9 (26.9–26.9)	30.4 (30.3–30.5)	< .0001
ALT^a^	17.5 (17.5–17.5)	31.2 (31.1–31.2)	29.8 (29.8–29.8)	29.5 (29.4–29.6)	< .0001
GGT^a^	18.9 (18.9–18.9)	51.0 (50.9–51.1)	43.8 (43.7–43.8)	68.5 (68.3–68.8)	< .0001

Data are presented as means ± standard deviations for continuous variables and *n* (%) for categorical variables

*Abbreviations: MAFLD* Metabolic-associated fatty liver disease, *BMI* Body mass index, *CCI* Charlson Comorbidity Index, *HDL-C* High-density lipoprotein cholesterol, *WC* Waist circumference, *eGFR* Estimated glomerular filtration rate, *TG* Triglyceride, *AST* Aspartate aminotransferase *ALT* Alanine aminotransferase, *GGT* Gamma-glutamyl transferase, *SBP* Systolic blood pressure, *DBP* Diastolic blood pressure

^a^Geometric means

### All-cause and disease-specific mortality by MAFLD subgroup

A total of 417,593 of the 9,935,314 subjects (4.2%) died during the median follow-up of 9.3 (interquartile range: 9.1, 9.6) years. Table 2 summarizes the observed associations between MAFLD subgroups and all-cause or cause-specific mortality. In the age- and sex-adjusted model, individuals in the MAFLD-diabetes group had the highest all-cause mortality among the four evaluated groups (HR: 1.43, 95% CI: 1.42–1.45). In the multivariable model adjusting for age, sex, BMI, income, smoking, alcohol consumption, exercise, and CCI score, individuals with MAFLD-diabetes had the highest increased risk of all-cause mortality (HR: 1.61, 95% CI: 1.59–1.63) followed by the MAFLD-lean and MAFLD-overweight/obese groups (HR: 1.36, 95% CI: 1.34–1.38 and HR: 1.19, 95% CI: 1.18–1.20, respectively, Table 2). When further adjusting for serum glucose, cholesterol, alanine aminotransferase, eGFR, liver cirrhosis, and hepatitis, these associations persisted. (Additional file 1: Table S1).

**Table 2:** All-cause and cause-specific mortality by MAFLD subgroup

	Death	Duration (PYs)	Incidence rate (per 1000 PY)	Hazard ratio (95% confidence interval)	
				Age, sex adjusted	Multivariate^a^
**All-cause mortality**					
No MAFLD	240,245	58,587,451.25	4.10	1 (Ref.)	1 (Ref.)
MAFLD-diabetes	58,797	4,848,887.35	12.13	1.43 (1.42, 1.45)	1.61 (1.59, 1.63)
MAFLD-overweight/obese	101,136	25,961,930.93	3.90	0.84 (0.84, 0.85)	1.19 (1.18, 1.20)
MAFLD-lean	17,415	1,727,529.60	10.08	1.45 (1.45, 1.49)	1.36 (1.34, 1.38)
**CVD-specific mortality**					
No MAFLD	45,670	58,587,451.25	0.78	1 (Ref.)	1 (Ref.)
MAFLD-diabetes	11,683	4,848,887.35	2.41	1.55 (1.51, 1.58)	1.61 (1.58, 1.65)
MAFLD-overweight/obese	19,718	25,961,930.93	0.76	0.94 (0.93, 0.96)	1.22 (1.19, 1.25)
MAFLD-lean	3191	1,727,529.60	1.85	1.51 (1.46, 1.57)	1.45 (1.40, 1.51)
**Cancer-specific mortality**					
No MAFLD	84,720	58,587,451.25	1.45	1 (Ref.)	1 (Ref.)
MAFLD-diabetes	19,822	4,848,887.35	4.09	1.37 (1.34, 1.39)	1.31 (1.29, 1.33)
MAFLD-overweight/obese	41,763	25,961,930.93	1.61	0.95 (0.94, 0.96)	1.11 (1.09, 1.13)
MAFLD-lean	5907	1,727,529.60	3.42	1.36 (1.32, 1.40)	1.27 (1.23, 1.30)
**Liver disease-related mortality**					
No MAFLD	12,824	58,587,451.25	0.22	1 (Ref.)	1 (Ref.)
MAFLD-diabetes	6626	4,848,887.35	1.37	2.99 (2.90, 3.08)	2.85 (2.75, 2.95)
MAFLD-overweight/obese	9535	25,961,930.93	0.38	1.28 (1.25, 1.32)	1.76 (1.70, 1.82)
MAFLD-lean	2305	1,727,529.60	1.33	3.24 (3.10, 3.39)	2.84 (2.72, 2.97)

*Abbreviations: MAFLD* Metabolic-associated fatty liver disease, *CVD* Cardiovascular disease, *PY* Person years

^a^Adjusted for age, sex, body mass index, income, smoking, alcohol consumption, exercise, and Charlson Comorbidity Index score

Overall, cancer and CVD represented the two most common causes of mortality in the current study; there were a total of 152,212, 80,262, and 31,290 cancer-associated, CVD-associated, and liver disease-associated deaths, respectively. In the multivariate model, individuals in the MAFLD-diabetes group had the highest CVD-specific mortality (HR 1.61, 95% CI: 1.58–1.65). Individuals in the MAFLD-lean and MAFLD-overweight/obese groups also had higher CVD-specific mortality than those without MAFLD (HR 1.45, 95% CI: 1.40–1.51 and HR 1.22, 95% CI: 1.19–1.25, respectively). Cancer-specific mortality increased significantly in all MAFLD subgroups compared to those without MAFLD. Individuals in the MAFLD-diabetes group showed the highest HR (1.31, 95% CI: 1.29–1.33), followed by the MAFLD-lean and MAFLD-overweight/obese groups (HR 1.27, 95% CI: 1.23–1.30 and HR 1.11, 95% CI: 1.09–1.13, respectively). Individuals in the MAFLD-diabetes group had a 2.85-fold higher liver disease-related mortality (HR: 2.85, 95% CI: 2.75–2.95), with similar risk for those in the MAFLD-lean group (HR: 2.84, 95% CI: 2.72–2.97), followed by those in the MAFLD-overweight/obese group (HR 1.76, 95% CI: 1.70–1.82), compared to those without MAFLD.

Because FLI has different cut-offs for men and women and bears a large gray zone of undetermined presence/absence of hepatic steatosis, we conducted analysis using two different sex-specific cut-offs [26, 27]. Similar findings were observed in the increase of HR according to the MAFLD subtypes (Additional file 1: Table S2).

To minimize the effect of age difference among the MAFLD subtypes, we performed sensitivity analysis according to subjects aged 60 years or older, and the results were similar to those of the entire population (Additional file 1: Table S3 and Table S4).

### Stratified analyses

We performed secondary analyses stratified by BMI. The increased risk for all-cause, cardiovascular, cancer, and liver-related mortality in the MAFLD-diabetes group was most prominent in individuals with underweight (*P* for interaction < 0.05). Liver-related mortality was the highest in MAFLD-lean individuals in the underweight group (HR, 5.03, 95% CI: 4.23–5.97, Table 3).

**Table 3:** Stratified analysis by body mass index: risk for all-cause and disease-specific mortality by MAFLD subgroup

BMI	MAFLD groups	Hazard ratio (95% confidence interval)			
		All-cause	Cardiovascular	Cancer	Liver-related
< 18.5	No MAFLD	1 (Ref.)	1(Ref.)	1 (Ref.)	1 (Ref.)
	MAFLD-diabetes	1.97 (1.79–2.15)	1.46 (1.13–1.89)	2.18 (1.81–2.61)	4.76 (3.88–5.83)
	MAFLD-overweight/obese	-	-	-	-
	MAFLD-lean	1.68 (1.56–1.81)	1.39 (1.13–1.71)	1.64 (1.41–1.90)	5.03 (4.23–5.97)
< 23	No MAFLD	1 (Ref.)	1(Ref.)	1 (Ref.)	1 (Ref.)
	MAFLD-diabetes	1.46 (1.43–1.49)	1.37 (1.30–1.43)	1.39 (1.34–1.44)	3.14 (2.96–3.31))
	MAFLD-overweight/obese	-	-	-	-
	MAFLD-lean	1.27 (1.25–1.29)	1.28 (1.23–1.33)	1.23 (1.20–1.27)	2.61 (2.49–2.75)
< 25	No MAFLD	1 (Ref.)	1(Ref.)	1 (Ref.)	1 (Ref.)
	MAFLD-diabetes	1.31 (1.29–1.34)	1.27 (1.22–1.33)	1.24 (1.20–1.28)	2.61 (2.44–2.78)
	MAFLD-overweight/obese	1.17 (1.15–1.18)	1.16 (1.13–1.20)	1.16 (1.04–1.19)	2.14 (2.03–2.25)
	MAFLD-lean	-	-	-	-
≥ 25	No MAFLD	1 (Ref.)	1 (Ref.)	1 (Ref.)	1 (Ref.)
	MAFLD-diabetes	1.21 (1.19–1.23)	1.24 (1.19–1.29)	1.13 (1.10–1.16)	3.18 (2.76–3.66)
	MAFLD-overweight/obese	1.13 (1.11–1.14)	1.16 (1.12–1.20)	1.09 (1.07–1.12)	1.75 (1.65–1.87)
	MAFLD-lean	-	-	-	-
*P* for interaction		< 0.001	0.031	< 0.001	< 0.001

Adjusted for age, sex, income, smoking, alcohol consumption, exercise, Charlson comorbidity index score, glucose, cholesterol, eGFR, systolic blood pressure, and waist circumference

*Abbreviations: MAFLD* Metabolic-associated fatty liver disease, *BMI* Body mass index, *eGFR* Estimated glomerular filtration rate

## Discussion

In this large nationwide, population-based cohort study, we calculated all-cause and disease-specific mortality in MAFLD subgroups using a database of health insurance claims in Korea. All-cause and disease-specific mortality were highest in the MAFLD-diabetes group, followed by the MAFLD-lean and MAFLD-overweight/obese groups, suggesting differential prognoses according to the MAFLD subtype. Interestingly, the liver-related mortality in the MAFLD-lean group was comparable to that in the MAFLD-diabetes group, emphasizing the poor liver-related prognosis in the MAFLD-lean group. The classification of MAFLD subtypes based on the metabolic phenotypes used in this study might help risk stratification of MAFLD patients.

Among the MAFLD subtypes, MAFLD-diabetes showed the highest increased risk for all-cause and disease-specific mortality. Diabetes is associated with a thrombotic and inflammatory condition and a higher incidence of atherosclerosis and carcinogenesis due to sustained hyperglycemia [28, 29]. As a consequence, diabetes has been recognized as a risk factor for all-cause and cause-specific mortality [30–32]. The results of this study showing that MAFLD-diabetes can be a strong predictor of all-cause and disease-specific mortality are in line with previous literature.

In addition to diabetes, MAFLD subcategorizations according to overweight/obesity may help elucidate the differential mortality risk among MAFLD subtypes. However, since the proportion of lean MAFLD is relatively low in real clinical practice and it is difficult to collect enough samples, the clinical and prognostic features of lean MAFLD have not been defined well in prior work. In the current study, although the prevalence of MAFLD-lean was 1.9% of the total population, the characteristics of the nationwide population study design enabled reliable analysis by securing a significant sample size.

In this study, individuals in the MAFLD-lean group were at a higher risk of all-cause and disease-specific mortality than those in the MAFLD-overweight/obese group. Similar to our results, several studies have reported that the cumulative CVD incidence rate was higher in lean MAFLD than in overweight/obese MAFLD [4, 19], and the risk of all-cause mortality was higher in those with lean MAFLD without diabetes (HR: 2.34) than in overweight/obese MAFLD subjects without diabetes (HR: 1.23) [10]. These findings are in line with poor clinical outcomes previously documented in patients with lean NAFLD [33–35]. A recent study reported that NAFLD subjects with normal BMI had a higher risk of all-cause mortality than those with obese NAFLD and that the major causes of death in this subgroup were cancer and CVD [36]. Similarly, people with lean NAFLD show a comparable risk of advanced liver disease, metabolic comorbidities, CVD, and liver-associated mortality to those with non-lean NAFLD without weight gain during follow-up and independent of genotype [37].

As lean NAFLD represents a unique subset related to the interaction of genetics, underlying medical conditions, and environmental exposures [38], the mechanism-related poor prognosis in lean MAFLD is not completely elucidated. Although lean and obese NAFLD share common altered metabolic and cardiovascular profiles, NAFLD patients having normal body weight show excess abdominal adipose tissue and metabolic abnormalities [39]. Recent studies reported that the progression of lean NAFLD is affected by multiple epigenetic mechanisms such as modification of cytosine and histones and changes in nucleosome localization [40], and circulating histones may discriminate the severity of steatosis in lean MAFLD patients [41]. In addition, a previous study reported that lean MAFLD subjects had lower fatty tissue index, lean tissue index, and total body water than did non-lean MAFLD subjects, which means that lean MAFLD subjects had less adiposity and less muscle mass compared to obese MAFLD subjects [42]. These results suggest that sarcopenia, which is the loss of muscle mass and function with aging, is a possible underlying reason for poor prognosis in people with lean MAFLD.

Because MAFLD is closely related to metabolic diseases, various pharmacological approaches that have been attempted to manage NAFLD’s metabolic traits, such as anti-obesity, antidiabetic, antioxidants, and cytoprotective agents, may also be effective in MAFLD. Emerging data from various clinical trials with drugs targeting diverse molecular mechanisms show promising results [43], and a recent phase II trial reported that semaglutide might be an attractive potential therapeutic option for NASH [44]. Future potential treatments for NAFLD include agents that act through peroxisomal proliferator-activated receptors, glucagon-like peptide-1 receptor agonists, sodium-glucose cotransporter 2 inhibitors, or farnesoid X receptor agonists, and several investigating agents have entered phase III clinical trials [45].

Overweight/obesity increases the risk for CVD, cancer, and liver-associated adverse outcomes. However, subjects in the MAFLD-overweight/obese group showed better prognoses than did those in the MAFLD-lean group in the present study. This phenomenon may be associated with the obesity paradox (i.e., elevated BMI may improve survival in individuals with CVD, primarily those with congestive heart failure [46, 47], cancer [48, 49], and type 2 DM [50]). Potential explanations for the obesity paradox include reverse causation, the inability to distinguish fat tissue from muscle mass using BMI, and even the possibility that adipose tissue may provide some level of protection. Although the evidence for an obesity paradox is mixed for individuals with a BMI between 22 and 24.9 kg/m^2^ [51, 52], the findings of increased mortality in individuals with lower BMI values are indisputable.

Our study presents the substantial strengths of a large sample size, a population-based design, and the categorization of MAFLD considering diabetes and metabolic risk factors in addition to BMI. This study provides new insights into the understanding of mortality-related outcomes of MAFLD, which can be used for risk stratification strategies in patients with MAFLD. However, we also acknowledge some limitations of the present work. First, although histological or radiological methods are more accurate for detecting hepatic steatosis than FLI, we used FLI as a surrogate marker of fatty liver and, thus, cannot accurately quantify the presence and severity of steatosis [53]. In addition, FLI has different cut-offs for men and women and bears a large gray zone of undetermined presence/absence of hepatic steatosis [26, 27]. However, similar results were observed when we used two different sex-specific cut-off values of FLI. Moreover, the use of FLI is practical for screening the general population in epidemiologic studies [10, 54], and FLI is regarded as an acceptable surrogate for diagnosing hepatic steatosis in MAFLD [55]. Second, our study cannot establish a causal relationship because of its population-based observational design. Third, there might be residual confounding factors such as family history or occupational exposures related to cancer development since the NHIS does not collect these data. Fourth, although most studies used BMI to define lean NAFLD/MAFLD [38], total body fat quantity (assessed based on BMI) does not reflect regional body fat distribution. Because visceral adiposity can be a target for interventions in the treatment of MAFLD, future studies need to consider visceral adiposity. Finally, it is possible that the evaluated conditions were under- or overestimated because the diagnoses were based on claims data (ICD-10 codes). However, the definitions we used in this study were validated in several previous studies [21, 22]. Further replicative research is warranted to validate and elucidate our results’ underlying mechanisms.

## Conclusions

Lean or diabetic MAFLD showed a poor prognosis for all-cause and disease-specific mortality. Classifying MAFLD subgroups based on metabolic phenotypes that account for integrated metabolic disorders might help risk stratification of patients with MAFLD.

## Supplementary Information

**Additional file 1: Table S1.** All-cause and cause-specific mortality by MAFLD subgroup.** Table S2.** All-cause and cause-specific mortality by MAFLD subgroup with sex-specific FLI cut-offs.** Table S3.** Baseline characteristics of the study population with aged ≥60 years.** Table S4.** All-cause and cause-specific mortality by MAFLD subgroup in individuals with aged ≥60 years.

## Data Availability

The dataset (NHIS) supporting the conclusions of this article is available in the homepage of National Health Insurance Sharing Service [http://nhiss.nhis.or.kr/bd/ab/bdaba000eng.do]. To gain access to the data, a completed application form, a research proposal, and the applicant’s approval document from the institutional review board should be submitted to and reviewed by the inquiry committee of research support in NHIS. Currently, the use of NHIS data is allowed only for Korean researchers.

## Abbreviations

BMI: Body mass index
CCI: Charlson Comorbidity Index
CI: Confidence interval
CVD: Cardiovascular disease
DM: Diabetes mellitus
eGFR: Estimated glomerular filtration rate
FLI: Fatty liver index
GGT: Gamma-glutamyl transferase
HR: Hazard ratio
ICD-10: International Classification of Diseases, 10th revision
MAFLD: Metabolic (dysfunction)-associated fatty liver disease
NAFLD: Nonalcoholic fatty liver disease
NHIS: National Health Insurance System
WC: Waist circumference
