# Supplementary material for: Lean or diabetic subtypes predict increased all-cause and disease-specific mortality in metabolic-associated fatty liver disease
Source: BMC Med. 2023 Jan 4;21:4. doi: 10.1186/s12916-022-02716-3 (PMC9814304; doi:10.1186/s12916-022-02716-3)
Supplement: Supplementary file 1 — Additional file 1: Table S1. All-cause and cause-specific mortality by MAFLD subgroup. Table S2. All-cause and cause-specific mortality by MAFLD subgroup with sex-specific FLI cut-offs. Table S3. Baseline characteristics of the study population with aged ≥60 years. Table S4. All-cause and cause-specific mortality by MAFLD subgroup in individuals with aged ≥60 years. [file 12916_2022_2716_MOESM1_ESM.docx]

**Table S1. All-cause and cause-specific mortality by MAFLD subgroup.**

|  | Death | | Duration (PYs) | Incidence rate (per 1,000 PY) | HR (95% CI) |
| --- | --- | --- | --- | --- | --- |
| **All- Cause mortality** |  | |  |  |  |
| No MAFLD | 240,245 | | 58587451.25 | 4.10 | 1(Ref.) |
| MAFLD-diabetes | 58,797 | | 4848887.35 | 12.13 | 1.40 (1.38,1.43) |
| MAFLD-overweight/obese | 101,136 | | 25961930.93 | 3.90 | 1.23 (1.21,1.24) |
| MAFLD-lean | 17,415 | | 1727529.60 | 10.08 | 1.39 (1.37,1.41) |
| **CVD-specific mortality** |  | |  |  |  |
| No MAFLD | 45,670 | | 58587451.25 | 0.78 | 1(Ref.) |
| MAFLD-diabetes | 11,683 | | 4848887.35 | 2.41 | 1.37 (1.34,1.41) |
| MAFLD-overweight/obese | 19,718 | | 25961930.93 | 0.76 | 1.23 (1.20,1.26) |
| MAFLD-lean | 3,191 | | 1727529.60 | 1.85 | 1.46 (1.41,1.51) |
| **Cancer-specific mortality** |  | |  |  |  |
| No MAFLD | 84,720 | | 58587451.25 | 1.45 | 1(Ref.) |
| MAFLD-diabetes | 19,822 | | 4848887.35 | 4.09 | 1.24 (1.22,1.27) |
| MAFLD-overweight/obese | 41,763 | | 25961930.93 | 1.61 | 1.14 (1.13,1.16) |
| MAFLD-lean | 5,907 | | 1727529.60 | 3.42 | 1.29 (1.26,1.33) |
| **Liver disease-related mortality** | |  |  |  |  |
| No MAFLD | 12,824 | | 58587451.25 | 0.22 | 1(Ref.) |
| MAFLD-diabetes | 6,626 | | 4848887.35 | 1.37 | 2.52 (2.43,2.62) |
| MAFLD-overweight/obese | 9,535 | | 25961930.93 | 0.38 | 1.95 (1.88,2.01) |
| MAFLD-lean | 2,305 | | 1727529.60 | 1.33 | 2.98 (2.85,3.12) |

**Abbreviations:** MAFLD, metabolic-associated fatty liver disease; CVD, cardiovascular disease; PY, person years; HR, hazard ratio; CI, confidence interval; ALT, alanine aminotransferase; eGFR, estimated glomerular filtration rate

Adjusted for age, sex, body mass index, income, smoking, alcohol consumption, exercise, Charlson comorbidity index score, glucose, cholesterol, ALT, eGFR, liver cirrhosis and hepatitis

**Table S2. All-cause and cause-specific mortality by MAFLD subgroup with sex-specific FLI cut-offs.**

|  | Hazard ratio (95% confidence interval) | | | | |
| --- | --- | --- | --- | --- | --- |
| FLI cut offs | Male 61.47, Female 51.65 | | | Male 31, Female 18 | |
| **All- Cause mortality** |  | | |  | |
| No MAFLD | 1(Ref.) | | | 1(Ref.) | |
| MAFLD-diabetes | 1.80 (1.78, 1.83) | | | 1.58 (1.57, 1.60) | |
| MAFLD-overweight/obese | 1.40 (1.38, 1.41) | | | 1.17 (1.16, 1.18) | |
| MAFLD-lean | 1.97 (1.91, 2.04) | | | 1.29 (1.27, 1.30) | |
| **CVD-specific mortality** |  | | |  | |
| No MAFLD | 1(Ref.) | | | 1(Ref.) | |
| MAFLD-diabetes | 1.74 (1.68, 1.79) | | | 1.59 (1.56, 1.63) | |
| MAFLD-overweight/obese | 1.39 (1.35, 1.42) | | | 1.19 (1.16, 1.21) | |
| MAFLD-lean | 1.83 (1.69, 1.99) | | | 1.40 (1.36, 1.45) | |
| **Cancer-specific mortality** |  | | |  | |
| No MAFLD | 1(Ref.) | | | 1(Ref.) | |
| MAFLD-diabetes | 1.44 (1.41, 1.48) | | | 1.28 (1.26, 1.31) | |
| MAFLD-overweight/obese | 1.22 (1.20, 1.25) | | | 1.11 (1.09, 1.12) | |
| MAFLD-lean | 1.58 (1.49, 1.68) | | | 1.18 (1.15, 1.21) | |
| **Liver disease-related mortality** | |  |  | |  |
| No MAFLD | 1(Ref.) | | | 1(Ref.) | |
| MAFLD-diabetes | 3.27 (3.14, 3.40) | | | 2.78 (2.68, 2.88) | |
| MAFLD-overweight/obese | 2.06 (1.99, 2.14) | | | 1.73 (1.67, 1.79) | |
| MAFLD-lean | 5.98 (5.60, 6.38) | | | 2.63 (2.51, 2.75) | |

**Abbreviations:** FLI, fatty liver index; MAFLD, metabolic-associated fatty liver disease; CVD, cardiovascular disease; PY, person years

*Adjusted for age, sex, body mass index, income, smoking, alcohol consumption, exercise, and Charlson comorbidity index score

**Table S3. Baseline characteristics of the study population with aged ≥60 years.**

|  | No MAFLD | MAFLD-diabetes | MAFLD-overweight/obese | MAFLD-lean | p-value |
| --- | --- | --- | --- | --- | --- |
|  | (n=1,236,075) | (n=228,610) | (n=589,573) | (n=53,736) |  |
| Age | 67.9 ± 6.3 | 67.6 ± 5.7 | 67.0 ± 5.6 | 67.9 ± 6.1 | <.0001 |
| Male | 516,872 (41.8) | 127,294 (55.68) | 310,105 (52.6) | 40,067 (74.56) | <.0001 |
| Income_low | 217,205 (17.6) | 40,756 (17.83) | 104,261 (17.68) | 10,091 (18.78) | <.0001 |
| Smoking |  |  |  |  | <.0001 |
| Non | 914,085 (74.0) | 147,445 (64.5) | 396,970 (67.3) | 25,177 (46.9) |  |
| Ex | 160,965 (13.0) | 43,395 (19.0) | 109,490 (18.6) | 11,371 (21.2) |  |
| Current | 161,025 (13.0) | 37,770 (16.5) | 83,113 (14.1) | 17,188 (32.0) |  |
| Alcohol consumption |  |  |  |  | <.0001 |
| Non | 947249 (76.6) | 151,302 (66.2) | 390,312 (66.2) | 24,466 (45.5) |  |
| Mild | 244,954 (19.8) | 58,203 (25.5) | 158,029 (26.8) | 19,553 (36.4) |  |
| Heavy | 43,872 (3.6) | 19,105 (8.4) | 41,232 (7.0) | 9,717 (18.1) |  |
| Regular exercise | 259,186 (21.0) | 50,216 (22.0) | 124,427 (21.1) | 10,414 (19.4) | <.0001 |
| Diabetes | 170,432 (13.8) | 228,610 (100.0) | 0 (0) | 0 (0) | <.0001 |
| Hypertension | 580,373 (47.0) | 174,650 (76.4) | 369,259 (62.6) | 32,674 (60.8) | <.0001 |
| Dyslipidemia | 312,721 (25.3) | 106,767 (46.7) | 209,051 (35.5) | 18,299 (34.1) | <.0001 |
| Hepatitis | 33,177 (2.7) | 8,511 (3.7) | 16,504 (2.8) | 1,580 (2.9) | <.0001 |
| Liver cirrhosis | 5,574 (0.5) | 2,252 (1.0) | 2,868 (0.5) | 555 (1.0) | <.0001 |
| CCI score |  |  |  |  | <.0001 |
| 0 | 554,739 (44.9) | 33,924 (14.8) | 268,179 (45.5) | 25,696 (47.8) |  |
| 1 | 308,824 (25.0) | 51,513 (22.5) | 158,129 (26.8) | 13,629 (25.4) |  |
| ≥2 | 372,512 (30.1) | 143,173 (62.6) | 163,265 (27.7) | 14,411 (26.8) |  |
| BMI | 22.6 ± 2.4 | 26.2 ± 2.9 | 26.6 ± 2.4 | 21.8 ± 1.1 | <.0001 |
| WC | 22.6 ± 2.4 | 26.2 ± 2.9 | 26.6 ± 2.4 | 21.8 ± 1.1 | <.0001 |
| SBP | 127.0 ± 16.0 | 132.8 ± 16.2 | 131.7 ± 15.7 | 132.8 ± 16.3 | <.0001 |
| DBP | 77.1 ± 10.0 | 79.5 ± 10.1 | 80.1 ± 10.0 | 80.5 ± 10.3 | <.0001 |
| Glucose | 99.6 ± 24.2 | 140.4 ± 45.0 | 97.3 ± 12.0 | 99.0 ± 12.6 | <.0001 |
| Total Cholesterol | 196.0 ± 43.8 | 198.0 ± 48.9 | 206.2 ± 44.2 | 204.3 ± 49.4 | <.0001 |
| HDL -C | 57.3 ± 38.2 | 51.4 ± 35.7 | 53.1 ± 37.5 | 55.6 ± 50.9 | <.0001 |
| eGFR | 81.7 ± 32.3 | 77.4 ± 31.8 | 79.5 ± 32.7 | 82.1 ± 36.4 | <.0001 |
| TG* | 98.7 (98.7-98.8) | 173.9 (173.6-174.3) | 160.5 (160.4-160.7) | 217.5 (216.6-218.3) | <.0001 |
| AST* | 23.6 (23.6-23.6) | 27.4 (27.3-27.4) | 26.2 (26.2-26.3) | 31.1 (30.9-31.2) | <.0001 |
| ALT* | 18.5 (18.5-18.5) | 26.9 (26.9-27.0) | 24.3 (24.3-24.3) | 26.3 (26.1-26.4) | <.0001 |
| GGT* | 20.0 (20.0-20.0) | 41.7 (41.6-41.9) | 34.8 (34.7-34.9) | 65.2 (64.8-65.7) | <.0001 |

NOTE: Data are presented as means ± standard deviations for continuous variables and n (%) for categorical variables.

Abbreviations: MAFLD, metabolic-associated fatty liver disease; CCI, Charlson comorbidity index; BMI, body mass index; WC, waist circumference; HDL-C, high-density lipoprotein cholesterol; SBP, systolic blood pressure; DBP, diastolic blood pressure; eGFR, estimated glomerular filtration rate; TG, triglyceride; AST, aspartate aminotransferase; ALT, alanine aminotransferase; GGT, gamma-glutamyl transferase

*Geometric means

**Table S4. All-cause and cause-specific mortality by MAFLD subgroup in individuals with aged ≥60 years.**

|  | Death | | Duration (PYs) | Incidence rate  (per 1,000 PY) | Hazard ratio (95% confidence interval) | |
| --- | --- | --- | --- | --- | --- | --- |
|  |  |  |  |  | Age, sex adjusted | Multivariate* |
| **All- Cause mortality** |  | |  |  |  |  |
| No MAFLD | 185,344 | | 10827281.83 | 17.12 | 1(Ref.) | 1(Ref.) |
| MAFLD-diabetes | 44,876 | | 1952148.13 | 22.99 | 1.37 (1.36, 1.39) | 1.57 (1.55, 1.59) |
| MAFLD-overweight/obese | 67,913 | | 5274231.64 | 12.88 | 0.83 (0.82, 0.83) | 1.19 (1.17, 1.20) |
| MAFLD-lean | 12,055 | | 450691.87 | 26.75 | 1.33 (1.31, 1.36) | 1.29 (1.26, 1.31) |
| **CVD-specific mortality** |  | |  |  |  |  |
| No MAFLD | 39,030 | | 10827281.83 | 3.60 | 1(Ref.) | 1(Ref.) |
| MAFLD-diabetes | 9,511 | | 1952148.13 | 4.87 | 1.46 (1.43, 1.50) | 1.56 (1.52, 1.60) |
| MAFLD-overweight/obese | 14,464 | | 5274231.64 | 2.74 | 0.88 (0.87, 0.90) | 1.18 (1.16, 1.21) |
| MAFLD-lean | 2,384 | | 450691.87 | 5.29 | 1.37 (1.31, 1.43) | 1.35 (1.30, 1.41) |
| **Cancer-specific mortality** |  | |  |  |  |  |
| No MAFLD | 60,645 | | 10827281.83 | 5.60 | 1(Ref.) | 1(Ref.) |
| MAFLD-diabetes | 14,765 | | 1952148.13 | 7.56 | 1.28 (1.25, 1.30) | 1.28 (1.25, 1.31) |
| MAFLD-overweight/obese | 27,703 | | 5274231.64 | 5.25 | 0.94 (0.93, 0.96) | 1.13 (1.11, 1.15) |
| MAFLD-lean | 4,355 | | 450691.87 | 9.66 | 1.35 (1.31, 1.39) | 1.25 (1.22, 1.29) |
| **Liver disease-related mortality** | |  |  |  |  |  |
| No MAFLD | 7,754 | | 10827281.83 | 0.72 | 1(Ref.) | 1(Ref.) |
| MAFLD-diabetes | 4,001 | | 1952148.13 | 2.05 | 2.60 (2.51, 2.71) | 2.50 (2.39, 2.62) |
| MAFLD-overweight/obese | 4,984 | | 5274231.64 | 0.94 | 1.27 (1.22, 1.32) | 1.70 (1.62, 1.78) |
| MAFLD-lean | 1,107 | | 450691.87 | 2.46 | 2.59 (2.43, 2.76) | 2.37 (2.22, 2.53) |

**Abbreviations:** MAFLD, metabolic-associated fatty liver disease; CVD, cardiovascular disease; PY, person years

*Adjusted for age, sex, body mass index, income, smoking, alcohol consumption, exercise, and Charlson comorbidity index score
